# Response: Commentary: Can Blood Flow Restricted Exercise Cause Muscle Damage? Commentary on Blood Flow Restriction Exercise: Considerations of Methodology, Application, and Safety

**DOI:** 10.3389/fphys.2020.574633

**Published:** 2020-10-30

**Authors:** Jamie F. Burr, Luke Hughes, Stuart Warmington, Brendan R. Scott, Johnny Owens, Takashi Abe, Jakob L. Nielsen, Cleiton Augusto Libardi, Gilberto Laurentino, Gabriel Rodrigues Neto, Christopher Brandner, Juan Martin-Hernandez, Jeremy Loenneke, Stephen D. Patterson

**Affiliations:** ^1^Human Performance and Health Research Laboratory, Department of Human Health and Nutritional Sciences, College of Biological Science University of Guelph, Guelph, ON, Canada; ^2^School of Sport, Health and Applied Science, St Mary's University, Twickenham, United Kingdom; ^3^School of Exercise and Nutrition Sciences, Institute for Physical Activity and Nutrition, Deakin University, Geelong, VIC, Australia; ^4^Murdoch Applied Sports Science Laboratory, Murdoch University, Murdoch, WA, Australia; ^5^Owens Recovery Science, San Antonio, TX, United States; ^6^Kevser Ermin Applied Physiology Laboratory, Department of Health, Exercise Science, and Recreation Management, University of Mississippi, Oxford, MS, United States; ^7^Department of Sports Science and Clinical Biomechanics, Faculty of Health Sciences, University of Southern Denmark, Odense, Denmark; ^8^Federal University of São Carlos, São Carlos, Brazil; ^9^School of Physical Education and Sport, University of São Paulo, São Paulo, Brazil; ^10^Faculdade de Enfermagem e Medicina Nova Esperança, Mossoró, Brazil; ^11^Aspire Academy for Sports Excellence, Doha, Qatar; ^12^Miguel de Cervantes European University, Valladolid, Spain

**Keywords:** occlusion, ischemia, hypoxia, BFR, muscle hypertrophy, safety

Blood flow restricted (BFR) exercise is increasingly popular for rehabilitative and adjunct strength training wherein the use of reduced loads may be beneficial (Slysz et al., 2015; Hughes et al., 2017). A majority of peer-reviewed evidence appears to support the relative safety of BFR exercise in a supervised research/rehabilitation setting. However, as with any exercise, there remains a possibility of adverse outcomes, and the interference of normal blood flow patterns could potentially affect this risk. While overwhelming data supports the vast majority of exercise to be beneficial for health, vigorous physical activity can acutely increase the risk of a serious adverse event in susceptible people (Franklin et al., 2020). In this context, we must be aware that the introduction of BFR can make otherwise low-intensity exercise considerably more stressful; hence our unambiguous call for careful screening of participants, and methodical progression of training volumes with specific attention to those performing unaccustomed exercise (Patterson et al., 2019). We appreciate the recently expressed opinion of Wernbom and colleagues on the risk of BFR (Wernbom et al., 2020), which in many ways reinforces our original perspective that these potential risks of BFR training must be respected, without assuming that the physiological response to BFR will be identical to that of exercise in the absence of BFR. However, we feel that their commentary fails to acknowledge that a majority of the arguments they raise are indeed already reflected in our original position stand- and that our original conclusions, which we stand by, were based on a breadth of literature rather than select studies that support any particular narrative.

In their editorial Wernbom and colleagues pose the question, “*Can Blood Flow Restricted Exercise Cause Muscle Damage?*” and we feel safe suggesting the consensus is “*yes*” it could. In fact, we have highlighted existing evidence demonstrating that rare adverse events have indeed occurred with BFR-RE and it is important to note that such things can happen with “traditional” exercise as well. We, thus, contend that it is the likelihood of such events occurring, and the risk that they pose, which is of greater concern to the current debate about the risk of muscle damage. While our group holds that evidence for an increased risk of a serious adverse event in the form of rhabdomyolysis in a normal training environment remains very low (0.07–0.2%) (Thompson et al., 2017; Patterson et al., 2019), Wernbom and colleagues extrapolate from two studies on unaccustomed subjects to suggest: “…the incidence rate of rhabdomyolysis after acute BFR-RE would be as high as 22 and 67%,” and the rate of exercise-induced myopathy would be 33 and 100%.” Firstly, we wish to highlight that these estimates are from studies with low subject numbers (Yasuda et al., 2015; Sieljacks et al., 2016; both *n* ≤ 10) and the apparently high rates in these small studies are driven by only a few extreme responders. In one of these studies, the evidence for rhabdomyolysis is extrapolated from blood measures collected on only 3 subjects (Yasuda et al., 2015). Taken out of context, it is perhaps difficult to fully comprehend these disparate estimates of risk, which exist at opposite ends of the spectrum. Despite their own initial presentation of these statistics, we agree with Wernbom et al. that these numbers cannot be taken to represent BFR in general- and for a number of reasons, which we expand upon to follow- these figures are misleading and support a counter-argument to a point that our group is not attempting to make. Despite taking a reductionist approach here for brevity of our response, we wish to highlight that both group's points are made with respect to nuance and circumstance; and the possibility that BFR related rhabdomyolysis *could* occur given the presence of multiple pre-disposing factors, vs. the *likelihood* of it occurring in a well-controlled setting, are perhaps very different arguments.

The largest discrepancy in this risk interpretation stems from the definition of meaningful muscle damage, and subsequently the occurrence of *bona fide* exertional rhabdomyolysis. Exertional rhabdomyolysis is a serious, or even fatal, condition associated with renal injury, and diagnosis depends on a combination of clinical expertise and laboratory findings that take into account participant pain, weakness, and muscle swelling, combined with substantially elevated levels of creatine kinase (CK) (Tietze and Borchers, 2014). Notably, our group embraces this strict definition of rhabdomyolysis, which we view as a serious adverse event, though we recognize that others may classify this on more of a continuum, which would affect the categorization of incidence. As Wernbom and colleagues themselves recognize, CK is an indirect measure of damage, and thus we suggest overreliance on this marker to interpret risk could be problematic. Elevated circulating CK following vigorous activity is common, and can occur in any individual who exercises beyond his or her normal muscular capabilities, often present in competitive athletes, deconditioned persons pushed far beyond their limits, and military recruits (Randall et al., 1996). Marathon runners, for example, demonstrate increases in circulating CK up to 25 times baseline following competition (Siegel et al., 1980); yet despite high CK, it is worth noting that the need for medical intervention remains low. Owing to the poorly defined cut-offs for laboratory values of CK, various thresholds have been suggested; however, these must be taken in context and applied judiciously. We suggest caution in interpreting evidence that all CK values >10,000 U/L are necessarily indicative of exertional rhabdomyolysis or that 2,000 U/L to be indicative of myothpathy as Wernbom and colleagues suggest, given that the original references for these indicators were not in the context of exercise, but rather considered renal consequences of statin therapy (Clarkson et al., 2006). While we can appreciate the desire to use an objective cut-off, we do not believe the literature currently provides sufficiently supported evidence of such ranges with specific reference to exertional rhabdomyolysis, and it is worth noting that some individuals could present with adverse responses at much higher or lower levels than others, depending on individual factors including hydration status, body mass, or renal function. As such, we believe greater weight should presently be given to evidence of actual adverse events, rather than a single risk factor/marker about which there still exists substantial debate regarding prognostic value.

Accepting that elevations in CK and rhabdomyolysis are not synonymous, the original work that Wernbom and colleagues highlight from their own group and others should be commended, as it adds to our understanding of muscular stress and potential for damage and adaptation interference during BFR exercise training (Sieljacks et al., 2016; Bjørnsen et al., 2019). While we did not cite some of this work in our earlier review owing to it being released after our publication, these studies clearly demonstrate the possible magnitude of CK changes following BFR use. Given the large interindividual difference in response to unaccustomed muscular work (Sieljacks et al., 2016), we believe this data further supports our original conclusion that certain individuals are more susceptible to muscular insult than others, and the use of particularly challenging BFR exercise in untrained participants demonstrates our point advocating for tailored individualized progression. We have also highlighted the fact that the well-accepted repeated bout effect, which protects against muscular soreness and damage in subsequent challenging exercise, appears to be applicable to BFR exercise in the same way it is for non-occluded exercise. Wernbom and colleagues, in their editorial, suggest BFR exercise led to greater muscle damage than that which was observed following eccentric exercise (Sieljacks et al., 2016). However, overall the indirect measures of muscle damage were comparable between BFR and maximal eccentric exercise, a key finding acknowledged in the original study. This suggests that the specific use of BFR exercise does not necessarily magnify risk compared to a more traditional (non-occluded) challenging exercise, which is an important benchmark in understanding the risk of BFR in relation to muscle damage. It is worth noting that in this specific study, BFR exercise was performed to task failure, which we have acknowledged likely increases the muscular stress of a given exercise bout and results in muscular adaptations, but is not required for most people. In fact, recently reported non-failure based BFR protocols from this very group present similar results as failure protocols (Sieljacks et al., 2019) which is in line with our recommendations. However, even when an acute bout of BFR exercise is performed to task failure, recent evidence would suggest that muscle damage is similar to high-load resistance training, which is traditionally recommended to promote neuromuscular adaptations (Alvarez et al., 2020).

Despite the claim by Wernbom and colleagues that all of the studies showing prominent alterations in CK/muscle damage (Yasuda et al., 2015; Sieljacks et al., 2016; Bjørnsen et al., 2019) were in line with the model of exercise prescription we suggest (see Table 1 in Patterson et al., 2019), we feel this to be somewhat disingenuous as participants in these studies were (by design) not accommodated to resistance exercise and not prescribed exercise in a progressive fashion. While these studies have adhered to some individual components of the suggested guidelines, it is worth noting that the combined prescription of cuff-pressure, repetitions, sets, and daily/weekly exercise frequency should be considered concurrently, as they are likely to interact. Prescribing BFR exercises that exist on the high-end of more than one of the recommended ranges across the exercise variables can make the overall stress of any given prescription much more intensive. As such, it is not surprising that this type of exercise would be challenging for previously unaccustomed participants, and the table recommendations must be considered in the overall context of our paper and the explicit guidelines that suggest an individually tailored, progressive approach. Even with these intensive exercise protocols, the elevation of CK and markers of muscular stress resulted in some expected perceived discomfort and muscle damage but did not lead to conditions requiring medical intervention. As the study investigators highlight, and as we noted in our original text, previous training further attenuated the rise in CK and other markers of muscle damage with a repeated bout (Sieljacks et al., 2016); and less intensive loading protocols resulted in a lesser response, thus supporting our recommendation for careful prescription to mitigate risk.

Ultimately, we agree with Wernbom and colleagues that BFR research remains in its infancy and further work to understand the extent of physiological systems affected, mechanisms of adaptation, and alterations in risk-stratification are required. While isolated case-reports demonstrating adverse reactions with the use of BFR exist in the literature, we contend that this represents a very small proportion of users, and many of the current cases can be explained by predisposing factors, or improper exercise prescription. Until a greater amount of objective risk-specific evidence is available, there may be value in extrapolating potential risk from more mechanistic studies, but we urge caution in the interpretation of these surrogate measures, which represent physiological stress, but not necessarily health risk.

## Author Contributions

All authors listed have made a substantial, direct and intellectual contribution to the work, and approved it for publication.

## Conflict of Interest

The authors declare that the research was conducted in the absence of any commercial or financial relationships that could be construed as a potential conflict of interest.
